# Depleted tumor suppressor miR-107 in plasma relates to tumor progression and is a novel therapeutic target in pancreatic cancer

**DOI:** 10.1038/s41598-017-06137-8

**Published:** 2017-07-18

**Authors:** Taisuke Imamura, Shuhei Komatsu, Daisuke Ichikawa, Mahito Miyamae, Wataru Okajima, Takuma Ohashi, Jun Kiuchi, Keiji Nishibeppu, Hirotaka Konishi, Atsushi Shiozaki, Ryo Morimura, Hisashi Ikoma, Toshiya Ochiai, Kazuma Okamoto, Hiroki Taniguchi, Eigo Otsuji

**Affiliations:** 1 Division of Digestive Surgery, Department of Surgery, Kyoto Prefectural University of Medicine, 465 Kajii-cho, Kawaramachihirokoji, Kamigyo-ku Kyoto, 602-8566 Japan; 2 Department of Surgery, Kyoto Second Red Cross Hospital, 355-5 Kamanzadoori Marutamachi Haruobicho, Kamigyo-ku 602-8026 Kyoto, Japan

## Abstract

This study explored decreased tumor suppressor microRNA (miRNA) plasma levels in pancreatic cancer (PCa) patients to clarify their potential as novel biomarkers and therapeutic targets. We used the microRNA array-based approach to select candidates by comparing plasma levels between PCa patients and healthy volunteers. Six down-regulated miRNAs (miR-107, miR-126, miR-451, miR-145, miR-491-5p, and miR-146b-5p) were selected. Small- and large-scale analyses using samples from 100 PCa patients and 80 healthy volunteers revealed that miR-107 was the most down-regulated miRNA in PCa patients compared with healthy volunteers (*P* < 0.0001; area under the receiver-operating characteristic curve, 0.851). A low miR-107 plasma level was significantly associated with advanced T stage, N stage, and liver metastasis and was an independent factor predicting poor prognosis in PCa patients (*P* = 0.0424; hazard ratio, 2.95). miR-107 overexpression in PCa cells induced G1/S arrest with the production of p21 and inhibited cell proliferation through the transcriptional regulation of Notch2. *In vivo*, the restoration and maintenance of the miR-107 plasma level significantly inhibited tumor progression in mice. Depletion of the tumor suppressor miR-107 in plasma relates to tumor progression and poor outcomes. The restoration of the plasma miR-107 level might be a novel anticancer treatment strategy for PCa.

## Introduction

Pancreatic cancer (PCa) is the fourth leading cause of cancer-related death in Japan and the United States^1^ and the seventh worldwide^2^. PCa is one of the most aggressive cancer types and constitutes a global health problem, with more than 330,000 cancer-related deaths annually^2^. Although perioperative chemo- and/or radiotherapy regimens, surgical techniques and perioperative management have greatly progressed, PCa continues to present an extremely poor prognosis. Even now, the median survival time of PCa patients is 5 to 8 months, and their 5-year survival rate is less than 10%. This is because PCa develops with no symptoms, local invasiveness, or metastases to distant organs in the early stage of its clinical course^3–5^. Therefore, novel early diagnostic tools and effective treatment strategies are urgently needed to improve the survival rate of PCa patients.

Because understanding the molecular mechanisms of tumorigenesis and identifying clinical biomarkers and molecular targets for PCa contribute to improving the management of this lethal disease, several studies have attempted to detect the biological factors involved in the malignant potential of PCa^6, 7^. Nevertheless, in clinical settings, no molecule has been used as an early diagnostic biomarker, and only a few molecules have been validated as therapeutic targets for PCa^8–10^. Hence, the development of novel molecular mechanisms and/or therapeutic targets is necessary to improve the prognosis of PCa.

MicroRNAs (miRNAs), which are small non-coding RNAs, regulate the translation of specific protein-coding genes. Since their discovery in 1993^11^, numerous studies have identified alterations in miRNA expression that are correlated with the progression of various diseases, including the development and progression of several cancer types^12–15^. In recent decades, several studies have elucidated in detail the mechanisms by which miRNAs are detectable in plasma/serum and are present in a remarkably stable form^13, 16–19^. Plasma/serum miRNAs are resistant to endogenous ribonuclease activity by binding to specific plasma proteins^20, 21^ or are packaged by various types of secretory vesicles, including apoptotic bodies and exosomes in plasma/serum^16, 22–24^. Furthermore, some extracellular miRNAs occur not only through cell lysis but also through active secretion^25–27^ and can function as intercellular transmitters^19, 26, 28, 29^. Thus, various blood-based miRNAs have been identified, including those in this study, and can be used for cancer detection, monitoring tumor dynamics, and predicting prognosis and chemoresistance^30–42^.

Recently, Kosaka and Ochiya *et al*. suggested a novel mechanism - that miRNAs could facilitate the system of maintenance and surveillance against cancer progression. Tumor suppressor miRNAs are normally secreted from neighboring healthy cells to cancer cells to inhibit cancer progression^43^. In our previous study, we identified that some tumor suppressor miRNAs in plasma, such as let-7a in gastric cancer^30^ and miR-375 in esophageal^32^ and pancreatic cancer^33^, were significantly down-regulated in cancer patients compared with healthy volunteers. As circulating miRNAs are considered to be released from cancer tissues as well as normal tissues, most of these miRNAs are expected to have originated from normal tissues; thus, we hypothesized that tumor suppressor miRNAs might become depleted from healthy cells in accordance with cancer progression. Indeed, we demonstrated that the low plasma level of tumor suppressor miR-375 in esophageal cancer patients was associated with worse survival^32^. Consequently, we suggested the novel theory that the down-regulation of some tumor suppressor miRNAs in the blood stream could be correlated with tumor progression and poor prognostic outcomes^32^.

In this study, we focused on tumor suppressor miRNAs that are down-regulated in PCa patient plasma, and we demonstrated the potential utility of the restoration of these tumor suppressor miRNAs as a therapeutic strategy for this lethal disease. We selected six down-regulated tumor suppressor miRNAs (miR-451, miR-126, miR-145, miR-146b-5p, miR-491-5p, and miR-107) in plasma through a comprehensive miRNA array-based approach. We finally validated that depleted tumor suppressor miR-107 plasma levels are related to tumor progression and poor outcomes. The restoration and maintenance of the miR-107 plasma level significantly inhibited tumor progression *in vivo*. Our results and concepts provide evidence that the restoration and maintenance of the tumor suppressor miR-107 plasma level could be a novel treatment strategy for PCa patients via nucleic acid medicine.

## Results

### Study design to find depleted tumor suppressor miRNAs in PCa patient plasma

**Figure 1:**
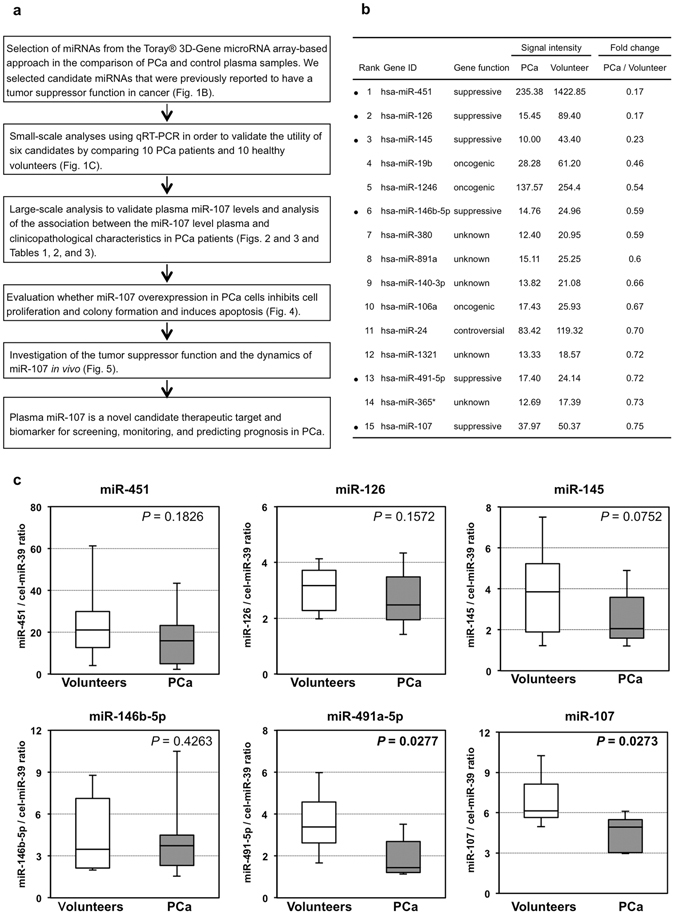
Study design and Selection of plasma miRNA candidates. (**a**) Study design to find novel candidate miRNAs that decreased in patient plasma as a therapeutic target for PCa. (**b**) Using a miRNA array-based approach, we found decreased plasma miRNAs with tumor suppressor functions by comparing the plasma levels of each miRNA between PCa patients and healthy volunteers. (**c**) Small-scale analysis of the plasma levels of six miRNAs in PCa patients and healthy volunteers by qRT-PCR. We investigated the plasma levels of the six selected miRNAs in 10 PCa patients and 10 healthy volunteers by qRT-PCR. The miRNA array-based approach showed that all six candidate miRNAs tended to be lower in the plasma of PCa patients than in that of healthy volunteers, and the change in the expression of miR-107 (*P* = 0.027) was found to be the most significant.

This study was designed as follows: (1) selection of appropriate miRNA candidates based on comparing plasma expression levels between PCa patients and healthy volunteers using the Toray^®^ 3D-Gene microRNA array-based approach; (2) small-scale analysis of plasma samples using qRT-PCR to validate the utility of the selected miRNA candidates; (3) large-scale analysis to validate the miR-107 plasma level and investigate how it is associated with clinicopathological characteristics and prognostic outcomes in PCa patients; (4) evaluation of whether miR-107 overexpression in PCa cells induces anti-tumor effects *in vitro*; and (5) investigation of the tumor suppressor function and dynamics of miR-107 *in vivo* (Fig. 1a).

### Selection of plasma miRNA candidates from the comprehensive miRNA array-based approach

We selected miRNA candidates using a miRNA array-based approach. These candidates had tumor suppressor functions and were decreased in patient plasma based on a comparison of the plasma levels of each miRNA between PCa patients and healthy volunteers (Fig. 1b). Of the 1719 candidates analyzed, in PCa patients, the expression levels of 15 plasma miRNAs were less than three-quarters those in healthy volunteers. Of these 15 miRNAs, we selected six miRNAs, miR-451, miR-126, miR-145, miR-146b-5p, miR-491-5p, and miR-107, which were previously reported to have a tumor suppressor role because our previous studies revealed that some tumor suppressor miRNAs in plasma were significantly down-regulated in cancer patients compared with healthy volunteers^30, 32, 33^, and the down-regulation of tumor suppressor miRNAs in the blood stream might be related to tumor progression and poor prognostic outcomes^32^.

### Small-scale analysis of the plasma levels of six miRNAs in PCa patients and healthy volunteers

First, we investigated the plasma levels of the selected six miRNAs in 10 PCa patients and 10 healthy volunteers by qRT-PCR using a small-scale analysis. As shown by the results of the miRNA array-based approach, the levels of all six candidate miRNAs tended to be lower in the plasma of PCa patients than in that of the healthy volunteers, and the difference in the expression level of miR-107 (*P* = 0.0273) was determined to be the most significant (Fig. 1c). Therefore, in this study, we selected miR-107 for further analyses.

### Large-scale analysis of the miR-107 plasma level in PCa patients

**Figure 2:**
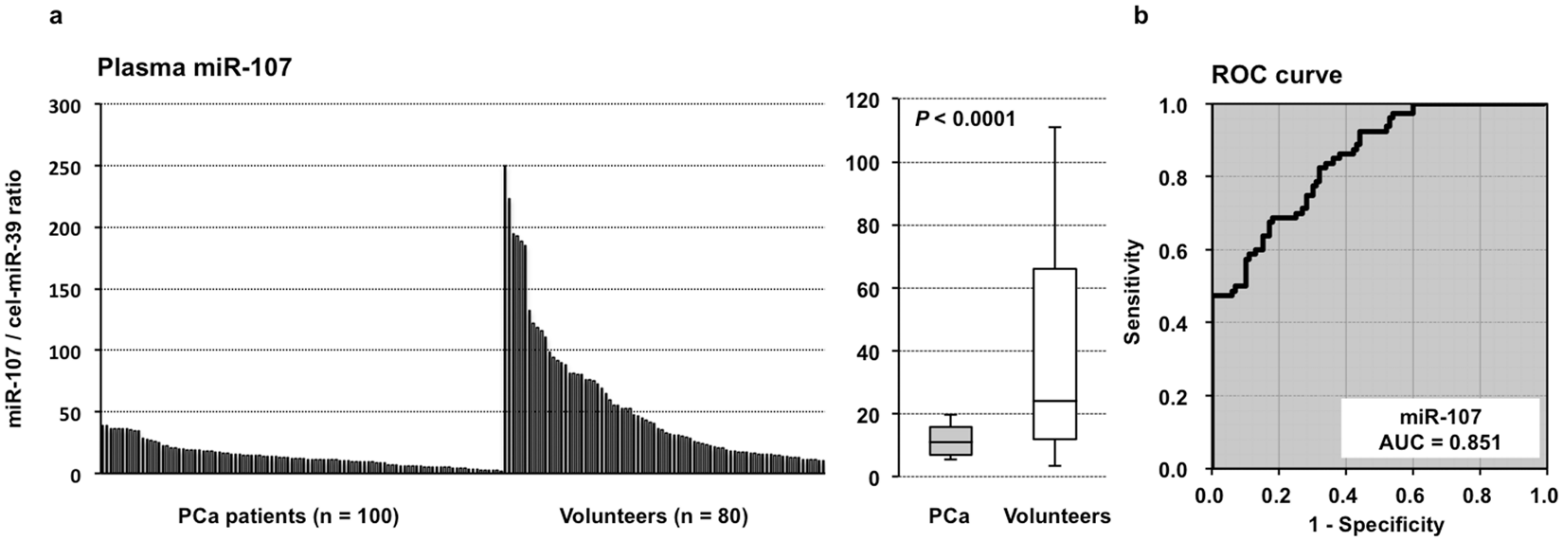
Large-scale analysis of the miR-107 plasma level in PCa patients and healthy volunteers. (**a**) We observed that the plasma level of miR-107 was significantly lower in PCa patients than in healthy volunteers (*P* < 0.0001). A waterfall plot also demonstrated a similar result (*P* < 0.0001). (**b**) Receiver-operating characteristic (ROC) curves and area under the ROC curve (AUC) values were used to assess the feasibility of using plasma miRNA levels as a diagnostic tool for detecting PCa. We calculated the AUC value to be 0.851. The optimal relative expression cut-off point was found to be 20.5 using the miR-107/cel-miR-39 ratio, with a sensitivity of 82.0%, a specificity of 68.8%, and an accuracy of 82.0%.

We next validated our observations in a large-scale setting. Plasma miR-107 was detectable in all samples from 100 PCa patients and 80 healthy volunteers. We observed that the miR-107 plasma level was significantly lower in the PCa patients than in the healthy volunteers (*P* < 0.0001). A waterfall plot also demonstrated a similar result (*P* < 0.0001) (Fig. 2a). Recent reports have demonstrated that some circulating miRNAs may be derived from peripheral blood cells^44^. To exclude the possible contamination of cellular RNA from peripheral blood cells, we examined the correlation between the plasma level of miR-107 and the number of any type of peripheral blood cells; no significant correlations were observed (Supplementary Figure S1). Furthermore, to assess the diagnostic ability of plasma miR-107 and to detect the optimal cut-off points that could differentiate cancer patients from healthy volunteers, we performed a receiver operating characteristic (ROC) analysis (Fig. 2b). The ROC curve was created by plotting the sensitivity against the false positive rate (1- specificity) at various threshold settings. We utilized the AUC value and the Youden index^45^ and found that the AUC value was 0.851. The optimal relative expression cut-off point was indicated to be 20.5, with a sensitivity of 82.0% and a specificity of 68.8%. Our results provide evidence that the miR-107 plasma level can be used to distinguish PCa patients from healthy volunteers to a clinically satisfactory degree in comparison with conventional tumor markers.

### Correlation between the miR-107 plasma level and clinicopathological factors in PCa patients

We analyzed the correlation between the miR-107 plasma level and clinicopathological factors in 74 PCa patients undergoing pancreatectomy (Table 1). The analysis was performed by dividing the patients into two groups using the median relative expression of miR-107, 10.29, in all PCa patients as a cut-off. A low miR-107 plasma level was significantly correlated with advanced N stages (*P* = 0.0376) and tended to be associated with advanced T stages (*P* = 0.0755). After pancreatectomy, patients with a low miR-107 plasma level more frequently developed recurrences (*P* = 0.0041), and especially liver metastases (*P* = 0.0027) (Table 2).

**Table 1:** Association between plasma miR-107 levels and clinicopathological characteristics in PCa patients with pancreatectomy.

n		Plasma miR-107 concentration				*P*-value^a^
		High		Low		
Total	74	50		24		
Age						0.7301
<65	22	15	(30%)	7	(29%)	
≥65	52	35	(70%)	17	(71%)	
Sex						0.3258
Male	34	21	(42%)	13	(54%)	
Female	40	29	(58%)	11	(46%)	
pT (TNM)						0.0755
T1-2	10	9	(18%)	1	(4%)	
T3-4	64	41	(82%)	23	(96%)	
pN (TNM)						**0.0376**
N0	31	25	(50%)	6	(25%)	
N1	43	25	(50%)	18	(75%)	
pM (TNM)						0.7386
M0	70	47	(94%)	23	(96%)	
M1	4	3	(6%)	1	(4%)	

^a^Chi-square test. NOTE: significant values are in bold.

**Table 2:** Association between plasma miR-107 levels and types of recurrence in PCa patients with pancreatectomy.

	n	Plasma miR-107 concentration				*P*-value^a^
		High		Low		
Total	38	20	(40%)	18	(75%)	**0.0041**
Hematogenous recurrence						
Liver	9	2	(4%)	7	(29%)	**0.0027**
Lung	4	3	(6%)	1	(4%)	0.7386
Bone	2	1	(2%)	1	(4%)	0.6024
Lymphatic recurrence	13	7	(14%)	6	(25%)	0.2546
Peritoneal recurrence	6	5	(10%)	1	(4%)	0.3636
Local recurrence	4	2	(4%)	2	(8%)	0.4545

^a^Chi-square test. NOTE: significant values are in bold.

### Investigation into whether miR-107 plasma levels reflect tumor dynamics in plasma, pancreatic tissue and exosomes

**Figure 3:**
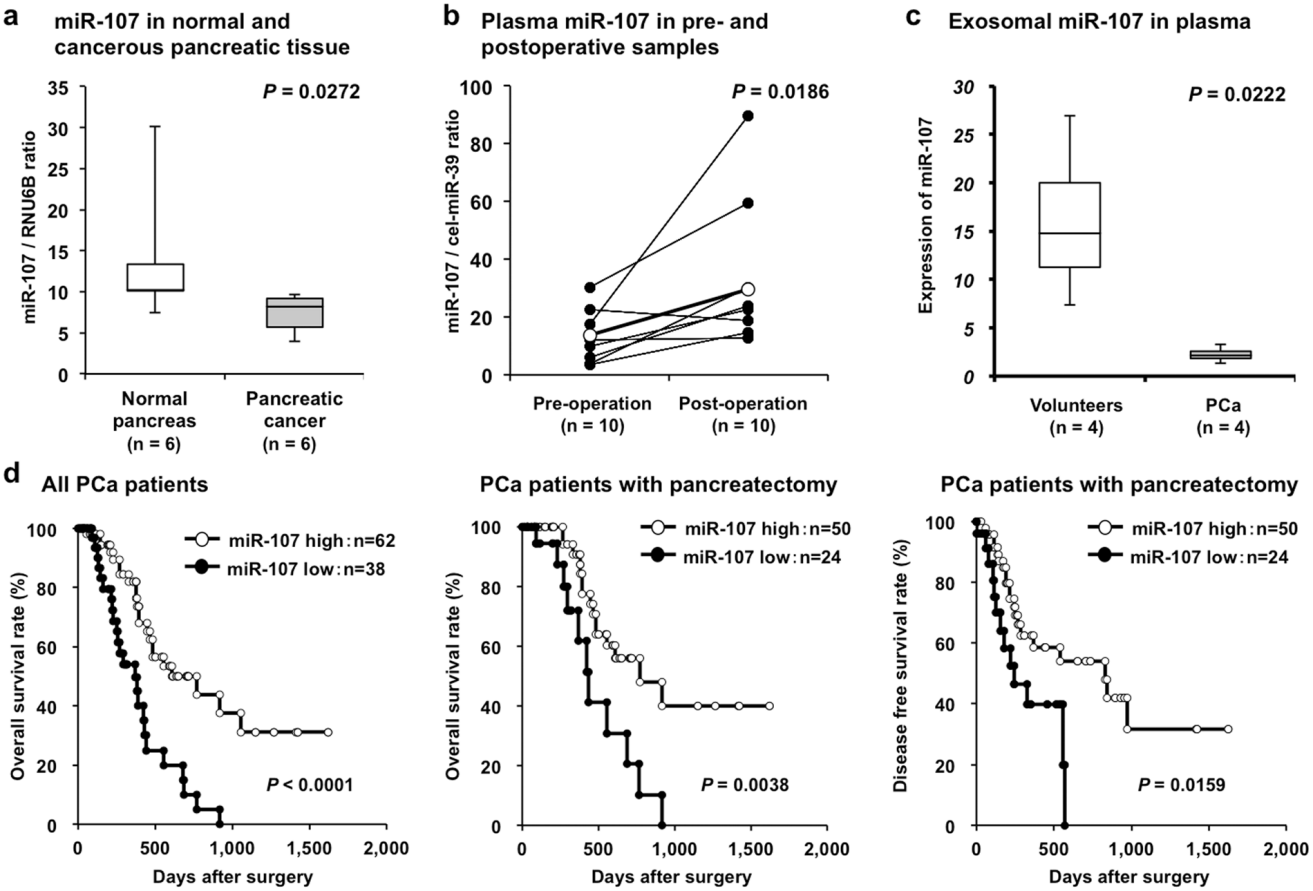
Investigation into whether plasma miR-107 levels reflect tumor dynamics in plasma, pancreatic tissue and exosomes. (**a**) Comparison of miR-107 expression between normal and cancerous pancreatic tissues. The expression of miR-107 was significantly higher in 6 normal pancreatic tissues than in 6 cancerous pancreatic tissues (*P* = 0.0272). (**b**) miR-107 plasma levels in pre- and postoperative samples from PCa patients who underwent curative pancreatectomy. miR-107 plasma levels in paired samples collected before and nearly 1 month after surgery from 10 PCa patients who underwent curative pancreatectomy. Plasma miR-107 was significantly elevated in the postoperative plasma samples (*P* = 0.0186). (**c**) miR-107 expression level in exosomes extracted from the plasma of PCa patients and healthy volunteers. The expression level of miR-107 in exosomes extracted from plasma was investigated in 4 PCa patients and 4 healthy volunteers. Exosomal miR-107 in plasma was significantly down-regulated in PCa patients compared with healthy volunteers (*P* = 0.0222). (**d**) Decreased plasma miR-107 is associated with prognostic outcomes in PCa patients. A prognostic analysis revealed that a low miR-107 plasma level was significantly associated with a worse cause-specific survival rate in all PCa patients (*P* < 0.0001), a worse cause-specific survival rate in PCa patients with curative pancreatectomy (*P* = 0.0038), and a worse disease-free survival rate in PCa patients with curative pancreatectomy (*P* = 0.0159).

To gain insight into whether miR-107 levels reflect tumor dynamics in pancreatic tissue and exosomes, we first compared the expression of miR-107 between 6 normal and 6 cancerous pancreatic tissues. The expression of miR-107 was significantly higher in normal pancreatic tissue than in cancerous pancreatic tissue  (P = 0.0272) (Fig. 3a). Second, we evaluated the miR-107 plasma level in paired samples that were collected before and nearly 1 month after surgery from 10 PCa patients who underwent curative pancreatectomy. miR-107 was significantly elevated in the postoperative plasma samples (*P* = 0.0186) (Fig. 3b). Finally, we compared the miR-107 expression levels in exosomes extracted from the plasma of 4 PCa patients and 4 healthy volunteers. As shown in Fig. 3c, the plasma level of exosomal miR-107 was significantly down-regulated in PCa patients compared with healthy volunteers (*P* = 0.0222). These results strongly suggested that miR-107 levels reflect tumor dynamics and that miR-107 may be incorporated into exosomes and released into the plasma.The subjects were selected randomly from the available samples.

### Potential utility of miR-107 as a prognostic biomarker in PCa patient plasma

Moreover, a prognostic analysis revealed that a low miR-107 plasma level was significantly associated with a worse overall survival rate in all PCa patients (*P* < 0.0001), a worse overall survival rate in PCa patients with curative pancreatectomy (*P* = 0.0038), and a worse disease-free survival rate in PCa patients with curative pancreatectomy (*P* = 0.0159) (Fig. 3d). Univariate and multivariate analyses using the Cox proportional hazards regression model revealed that a low miR-107 level (*P* = 0.0424; hazard ratio, 2.95; 95% confidence interval [CI]: 1.03–9.46) and stage M1 disease (*P* = 0.0149; hazard ratio, 7.68; 95% CI: 1.58–30.0) were independent factors predicting poor prognosis in PCa patients (Table 3).

**Table 3:** Univariate and multivariate analyses of PCa patient survival following pancreatectomy using the Cox proportional hazards model.

	Variable	Univariate^a^	Multivariate^b^		
		*P*-value	HR	95% CI	*P*-value
Sex	Male vs. Female	0.5704		—	
Age	≥65 vs. <65	0.2171		—	
T stage (TNM)	T3-T4 vs. T1-T2	**0.0306**	2.06	0.52–10.0	0.3032
N stage (TNM)	N1 vs. N0	**0.0147**	1.00	0.30–3.26	0.9956
M stage (TNM)	M1 vs. M0	**0.0019**	7.68	1.58–30.0	**0.0149**
Plasma miR-107 expression	Low vs. High	**0.0038**	2.95	1.03–9.46	**0.0424**

^a^Kaplan–Meier method; significance was determined by the log-rank test. ^b^Multivariate survival analysis was performed using Cox’s proportional hazards model. HR: hazard ratio; CI: confidence interval. NOTE: significant values are in bold.

### Investigation of the tumor suppressor function of miR-107 in PCa cells

**Figure 4:**
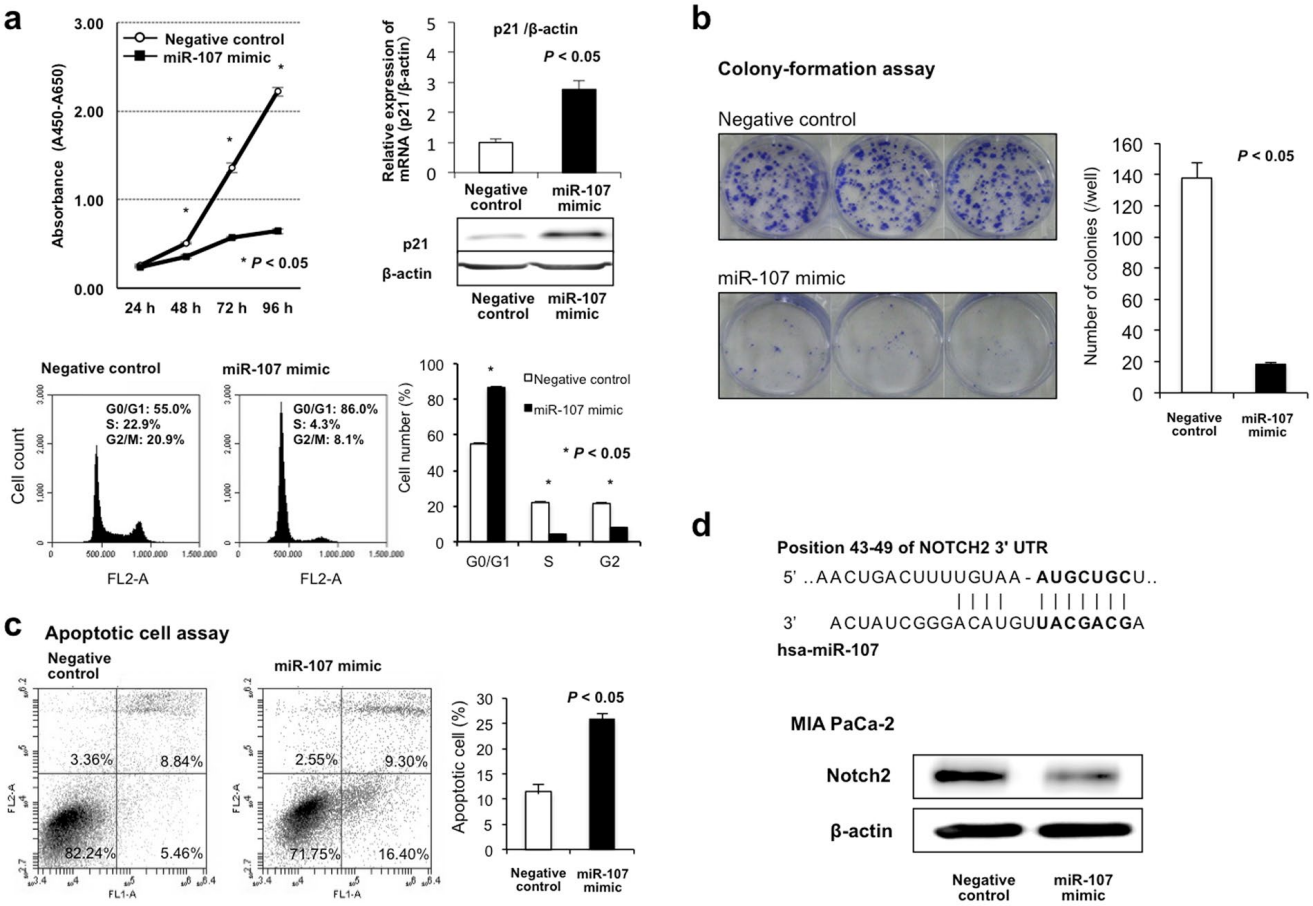
Investigation of the tumor suppressor function of miR-107 in PCa cells. (**a**) Cell proliferation analysis by miR-107 overexpression. Proliferation was significantly suppressed in MIA PaCa-2 cells transfected with the miR-107 mimic compared with cells transfected with the negative control mimic. The FACS analysis demonstrated that transfecting MIA PaCa-2 cells with the miR-107 mimic resulted in an accumulation of cells in the G0–G1 phase compared with transfection with the control miRNA mimic. In MIA PaCa-2 cells, p21 mRNA and protein levels were increased at 72 h after transfecting the cells with the miR-107 mimic. These findings indicated that miR-107 overexpression in PCa cells induced the production of p21, which results mainly in G0–G1 arrest. (**b**) miR-107 overexpression inhibits colony formation. Colony-formation assays were performed using MIA PaCa-2 cells. PCa cells were transiently transfected with the miR-107 or control mimic for two weeks. The number of colonies in MIA PaCa-2 cells treated with the miR-107 mimic was significantly lower than that in MIA PaCa-2 cells treated with the control mimic. (**c**) miR-107 overexpression induces cell apoptosis. The apoptotic cell analysis showed that miR-107 overexpression increased early apoptosis (annexin V-positive/PI-negative) and late apoptosis (annexin V/PI-double positive) 72 h after miR-107 mimic transfection compared with control mimic transfection in MIA PaCa-2 cells. (**d**) Notch2 as a novel target oncogene of miR-107 in PCa cells. An *in silico* search (http://www.targetscan.org/) identified Notch2 as a novel target oncogene of miR-107 in PCa. The seed regions of the miR-107 and complementary *Notch2* 3′UTR sequences are presented in this figure. miR-107 overexpression inhibited Notch2 protein production. Error bars indicate standard error of the mean (s.e.m.); n = 4 technical replicates of a representative experiment (out of four experiments).

To investigate the tumor suppressor function of miR-107 in PCa cells, we first performed a cell proliferation assay using miRNA mimics to investigate whether miR-107 overexpression would suppress PCa cell proliferation. Proliferation was significantly suppressed in all four PCa cell lines after miR-107 mimic transfection compared with negative control mimic transfection (MIA PaCa2 in Fig. 4a; the other cell lines in Supplementary Figure S2). The FACS analysis revealed that transfecting PCa cells with the miR-107 mimic induced the accumulation of G0–G1 phase cells compared with negative control mimic transfection (MIA PaCa2 in Fig. 4a; the other cell lines in Supplementary Figure S2). In all PCa cell lines, p21 mRNA and protein levels were increased at 72 h after miR-107 mimic transfection. To confirm the inhibitory effect of miR-107 against anchorage-independent cell growth, colony-formation assays were performed using MIA PaCa-2 cells. These PCa cells were transiently transfected with the miR-107 or control mimic for two weeks. The number of colonies was significantly lower in MIA PaCa-2 cells treated with the miR-107 mimic than in MIA PaCa-2 cells treated with the control mimic (Fig. 4b). The apoptotic cell analysis showed that miR-107 overexpression in MIA PaCa-2 cells increased early apoptosis (annexin V-positive/PI-negative) and late apoptosis (annexin V/PI-double positive) 72 h after miR-107 mimic transfection compared with control mimic transfection (Fig. 4c).

Finally, to investigate whether miR-107 directly regulates novel target oncogenes, we focused on the Notch2 gene, which has been reported to have oncogenic functions^46–52^ and was selected as a putative target using TargetScan (http://www.targetscan.org/). The seed regions of the miR-107 and complementary *Notch2* 3′UTR sequences are presented in Fig. 4d. miR-107 overexpression inhibited the production of Notch2 protein (MIA PaCa2 cells in Fig. 4d; the other cell lines in Supplementary Figure S2). These findings suggested that Notch2 is a candidate direct target of miR-107.

### Restoration and maintenance of the miR-107 plasma level could suppress tumor growth ***in vivo***

**Figure 5:**
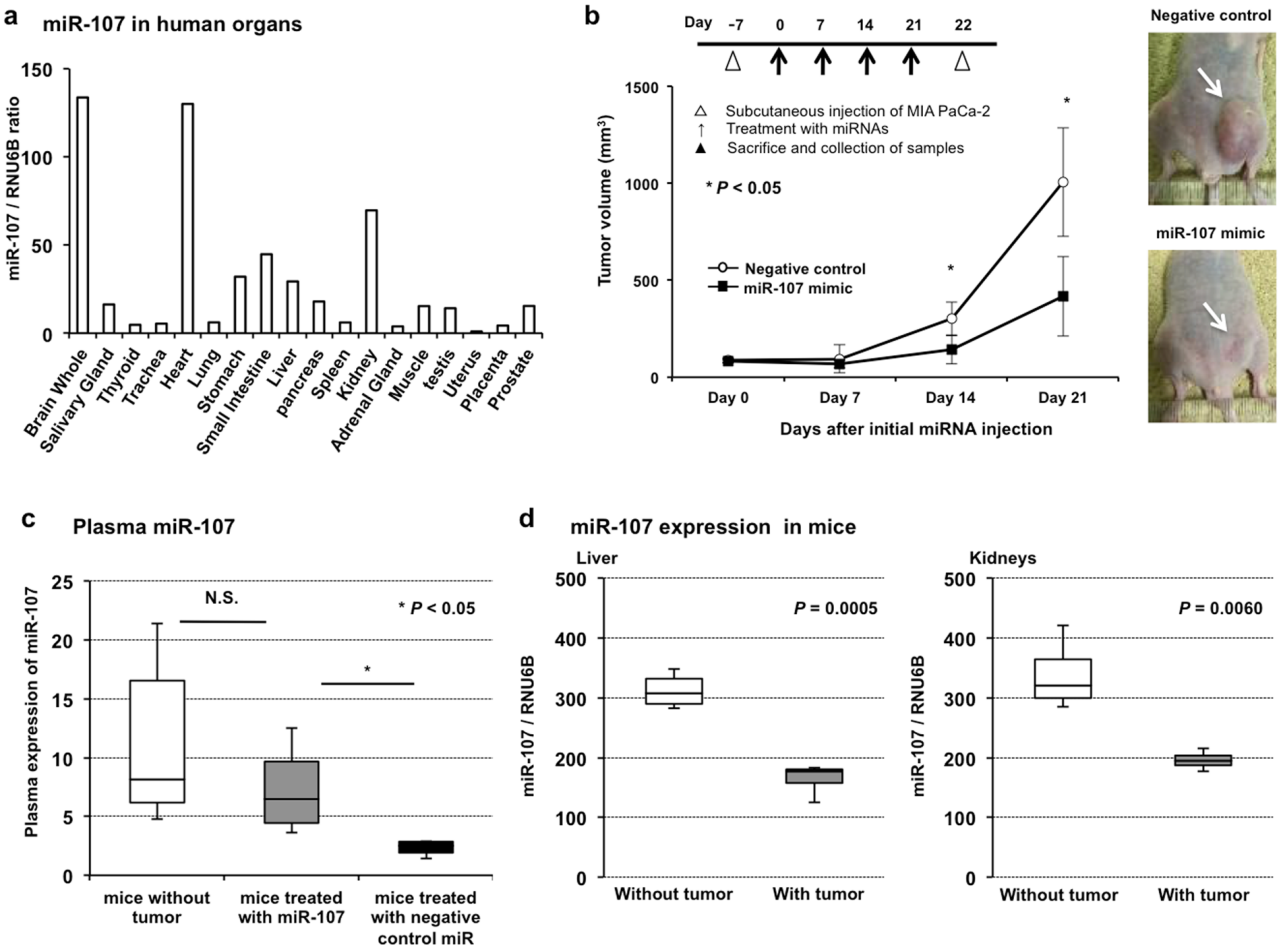
Restoration and maintenance of the miR-107 plasma level could suppress tumor growth *in vivo*. (**a**) Expression profiles of miR-107 in human organs. To gain insight into the systemic secretion of miR-107 from normal healthy cells, we examined the expression profiles of miR-107 in human organs. We used qRT-PCR to determine the expression of miR-107 in human tissues using a Human Total RNA Master Panel (Clontech Laboratories, Inc.). The expression of miR-107 was comparatively high in the brain, heart, kidneys, small intestine, stomach and liver. (**b**) Investigation into whether miR-107 could suppress tumor growth *in vivo*. To evaluate the tumor suppressor function of miR-107, the subcutaneous injection of the miR-107 or negative control mimic with atelocollagen around the tumor was repeated once a week for 4 weeks after the initial treatment. The miR-107 mimic significantly suppressed tumor growth compared with the negative control mimic. Error bars indicate s.e.m; n = 4 mice per group. (**c**) Investigation into the correlation between miR-107 expression in plasma and tumor growth suppression. miR-107 expression was measured in the plasma of tumor-bearing mice treated with miR-107 or the negative control mimic and mice without tumors. The miR-107 plasma level was significantly down-regulated in mice treated with the negative control mimic compared with mice without tumors, and the miR-107 plasma level in mice treated with miR-107 was preserved near that in mice without tumors. n = 4 mice per group. (**d**) Altered miR-107 expression in the liver and kidneys according to the presence of a tumor. In the liver and kidneys, the expression of miR-107 was significantly lower in tumor-bearing mice than in mice without tumors. In contrast, in muscle, the expression of miR-107 was significantly higher in tumor-bearing mice than in mice without tumors. n = 4 mice per group.

To gain insight into the systemic secretion of miR-107 from normal healthy cells, we examined the expression profiles of miR-107 in human organs. We used qRT-PCR to determine the expression of miR-107 in human tissues using a Human Total RNA Master Panel (Clontech Laboratories, Inc.). The expression of miR-107 was comparatively high in the brain, heart, kidneys, small intestine, stomach and liver (Fig. 5a).

Next, we examined the possible tumor suppressor function of miR-107 *in vivo* using miRNA mimics and SCID mice with subcutaneous tumors. Seven days after the subcutaneous injection of MIA PaCa-2 cells, tumor development at the injection site was visually confirmed. The miR-107 or negative control mimic with atelocollagen was subcutaneously injected around the tumor weekly for four weeks after the initial treatment (Fig. 5b). Compared with the negative control mimic, the miR-107 mimic more significantly suppressed tumor growth (Fig. 5b). The miR-107 plasma level was significantly lower in control mice treated with the control mimic than in mice treated with the miR-107 mimic. In contrast, the miR-107 plasma level in mice treated with the miR-107 mimic remained at a normal level and was not significantly different from that in non-treated mice without tumors (Fig. 5c). These findings strongly suggested that the restoration of miR-107 in plasma could significantly inhibit PCa tumor growth.

Furthermore, the expression of miR-107 was comparatively high in the liver and kidneys (Fig. 5a), tissue samples of which were easy to obtain; in addition, the expression levels of miR-107 were significantly lower in tumor-bearing mice than mice without tumors (Fig. 5d). These results may be applied to clarify the mechanism of plasma miR-107 depletion in PCa patients.

## Discussion

Numerous circulating miRNAs have been identified as potential biomarkers of various cancer types^16, 19, 25, 39, 53–55^. Regarding PCa, to date, more than thirty miRNAs have been reported as potentially useful biomarkers^31, 33, 37, 56–59^. Through our novel screening strategy using genome-wide miRNA microarray analyses to detect depleted tumor suppressor miRNAs in the plasma of PCa patients, we identified a novel plasma miRNA, miR-107, which has tumor suppressor functions and was significantly depleted in the plasma of PCa patients compared with healthy volunteers. The down-regulation of plasma miR-107 was shown to be related to tumor progression and worse outcomes in PCa patients. Moreover, *in vivo* analyses showed that injecting miR-107 around established PCa tumors enabled the restoration of the miR-107 plasma level to normal levels and induced significant tumor regression compared with the controls. These findings strongly suggest that the miR-107 plasma level could be a novel biomarker for cancer detection, disease monitoring, and predicting prognosis in PCa patients. Additionally, the depletion of plasma miR-107 could be a therapeutic target, and the restoration and maintenance of the tumor suppressor miR-107 plasma level could be a novel treatment strategy for PCa patients via nucleic acid medicine.

Concerning the molecular functions of miR-107, various biological functions in human physiological systems, such as the central nervous system^60–62^, the circadian system^63^, angiogenesis^64^, lipid metabolism^65, 66^, glycometabolism^67^ and inflammation^68^, have been reported. Regarding the function of miR-107 in cancer, reduced miR-107 expression has been reported in various types of cancer^69–78^, and several recent studies have identified the tumor suppressor functions of miR-107. One crucial function of miR-107 that has been identified is the inhibition of oncogenes, such as CDK8 in breast cancer^71^, the ATR/Chk1 pathway in cervical cancer^79^, HIF-1, CCND1, and NFKB1 in colon cancer^80, 81^, CDK in gastric cancer^70^, CDK6, Notch-2, and VEGF in glioma^74, 75, 82, 83^, CDK6 and CDK8 in non-small cell lung cancer^84, 85^, GRN in prostate cancer^78^, eukaryotic translation initiation factor 5 in renal clear cell carcinoma^86^, and CDK6 in PCa^87^. Furthermore, some studies have demonstrated that the tumor suppressor p53 inhibits tumor angiogenesis and cell growth through the transcriptional regulation of miR-107 in several cancer types^74, 80^. These previous results support the tumor suppressor role of miR-107 in PCa demonstrated in our study. In this study, we focused on Notch2, which has been reported as a target molecule for the treatment of PCa^88^ based on previous reports associated with tumorigenesis^46–48^ and malignant behavior in PCa^49–52^, and we first identified Notch2 as a possible direct target of miR-107 in PCa. Our findings suggest the possibility of miR-107 as a novel promising therapeutic target associated with the regulation of Notch2 overexpression in PCa.

The dynamics and origin of miR-107 circulating in the human body have not yet been elucidated. Thus, based on previous studies, including our own regarding the biology of circulating miRNAs^20, 22, 32^, we hypothesized that healthy cells may secret miR-107 packaged in exosomes into the blood stream and that the exosomes containing miR-107 are delivered via the blood stream to PCa cells. Then, miR-107 taken up by recipient cells might serve as an anti-tumor molecule. During the initial stage of tumorigenesis, the consumption of tumor suppressor miRNAs in cancer cells may be compensated for by the surrounding healthy cells, which supply exosomes containing tumor suppressor miRNAs. However, once the surrounding cells can no longer meet this demand, cancer cells progress to an advanced stage. Indeed, we demonstrated that the tumor suppressor miR-107 was incorporated into exosomes and that exosomal miR-107 was decreased in PCa patient plasma compared with that of healthy volunteers (Fig. 3d). In addition, a low miR-107 plasma level was related to nodal metastasis, advanced depth of invasion and recurrence. Namely, the decrease in the plasma level of miR-107 was strongly associated with disease progression and worse prognosis in PCa patients. Furthermore, *in vivo*, the restoration and maintenance of the miR-107 level in plasma significantly inhibited tumor progression in mice. These findings strongly suggest that during the initial stage of tumourigenesis, the down-regulation of tumor suppressor miRNAs in cancer cells may be compensated for by the surrounding healthy cells, which supply exosomes containing tumor suppressor miRNAs. However, once the surrounding cells can no longer meet this demand, cancer cells progress to an advanced stage.

Attentions have been paid by various researchers over the past few years to the development of miRNA-based medicine, and two promising studies have been performed toward this end. The first study focused on the therapeutic silencing of disease-associated miRNAs using miRNA inhibitors. Miravirsen (Santaris Pharma) is one of several promising miRNA inhibitors; it can bind to miR-122 and inhibit its biogenesis. Miravirsen was developed for the treatment of hepatitis C and is currently under evaluation in clinical trials^89–91^. The second study focused on therapeutic miRNA-based drugs using synthetic miRNA mimics. Recently, a phase I clinical trial using the miRNA mimic MIRX34 (Mirna Therapeutics, Inc.) was performed^92^. MIRX34 is a synthetic miRNA mimic of the tumor suppressor miR-34 and was administered to patients with primary or metastatic liver cancer. Unfortunately, this trial was ended because of serious adverse immune-related effects. As shown in this study, the administration of tumor suppressor miRNA mimics still carries the potential risk of inducing unexpected physiological adverse effects because miRNAs can regulate multiple genes affecting various biological functions. In this study, we focused on tumor suppressor miRNAs depleted in PCa patients and demonstrated that the restoration of the plasma miR-107 level to the normal physiological level might be a novel anticancer treatment for PCa. We believe that the restoration of tumor suppressor miRNAs, which are abundantly detected in the plasma of healthy individuals, may be a novel strategy for minimizing various physiological risks in clinical applications.

This is the first report to demonstrate that the tumor suppressor miR-107, which was depleted in the plasma of PCa patients and detected by comprehensive miRNA microarray analyses, could be a plasma biomarker as well as a therapeutic target for PCa. However, many issues must still be addressed before these findings can be translated into a clinically useful biomarker and treatment agent for PCa patients. Detailed examinations of the physiological effects of miR-107 are needed for its safe clinical utilization. Moreover, further studies are needed on the cellular uptake or secretion systems of tumor suppressor miRNAs, leading to the development of miRNA delivery systems for future therapeutic and diagnostic applications^93–96^. These studies are currently under evaluation. Furthermore, we believe that tumor suppressor miRNAs with more powerful anticancer effects could be identified from examining tumor suppressor miRNAs depleted in the plasma of patients with various cancer types using different strategies, such as next-generation sequencing or digital PCR-based approaches. These strategies are currently under evaluation and are expected to be reported upon in the near future.

## Methods

### Patients and samples

All experimental methods were carried out in accordance with relevant guidelines and regulations. Written informed consent was obtained from all patients to use their tissue specimens for research purposes, and the study was approved by the institutional review boards of Kyoto Prefectural University of Medicine and Kyoto Second Red Cross Hospital. Between January 2010 and April 2014, a total of 100 plasma samples from PCa patients and 80 samples from healthy volunteers were collected. The one hundred plasma samples from PCa patients consisted of 10 small-scale samples from the Kyoto Prefectural University of Medicine, 57 validation samples from the Kyoto Prefectural University of Medicine (1st cohort), and 33 validation samples from the Kyoto Second Red Cross Hospital (2nd cohort). All patient characteristics are presented in Supplementary Table S1. The eighty samples from healthy volunteers included those from medical personnel and patients with benign disease, such as cholecystolithiasis and inguinal herniation. These patients underwent medical examinations, including computed tomography and endoscopy, and were shown not to have any pancreatic or cancerous diseases. Tumor stages were assessed according to the Union for International Cancer Control classification system^97^.

Peripheral blood (7 ml) was obtained from each patient at the time of diagnosis or before surgery and from the healthy volunteers. The blood was transferred into sodium heparin tubes (BD Vacutainer, Franklin Lakes, NJ) and immediately subjected to the three-spin protocol (1500 r.p.m. for 30 min, 3000 r.p.m. for 5 min, and 4500 r.p.m. for 5 min) to prevent contamination by cellular nucleic acids. Plasma was collected and then stored at −80 °C until further processing. Histological evaluations were performed for tissues adjacent to specimens, according to the criteria of the World Health Organization. In all cases, two pathologists agreed with the pathological observations and confirmed the diagnosis.

### RNA extraction

Total RNA was extracted from 400 μl of plasma using a mirVana PARIS Kit (Ambion, Austin, TX) and finally eluted into 100 μl of preheated (95 °C) Elution Solution according to the manufacturer’s protocol. The reason why the volume of 400 μl of plasma was used as the common denominator in each microarray analysis is that there was no definite internal control in the plasma miRNA analyses, as shown in our previous studies^30–35, 98^. Total RNA was also extracted from four 15-μm-thick slices of formalin-fixed and paraffin-embedded tissue (for a total of 60 μm in thickness) using a RecoverAll Total Nucleic Acid Isolation Kit (Ambion) and then eluted into 60 μl of Elution Solution according to the manufacturer’s protocol.

### miRNA microarray analyses

Microarray analyses of the plasma samples were performed using the 3D-Gene miRNA microarray platform (Toray Industries, Kamakura, Japan)^35, 98–100^. The results were compared between the PCa patient samples and the healthy volunteer samples. Each 100-μl plasma sample from the three PCa patients, who underwent curative surgery, was equally mixed, and the total plasma volume of 300 μl was used as the PCa patient sample. On the other hand, each 100-μl plasma sample from the three healthy volunteers was equally mixed, and the 300 μl of plasma was used as the healthy volunteer sample. The RNA extraction and microarray analysis were performed according to the manufacturer’s instructions, as previously described^98^. In brief, the amount of total RNA in the plasma was too small; thus, 2 of 4 μl of the extracted total RNA from the 300-μl plasma samples were used in the microarray experiments. This RNA was labeled with Hy5 using the Label IT miRNA Labeling Kit (Takara Bio, Otsu, Japan) and hybridized at 32 °C for 16 h on a 3D-Gene chip. The 3D-Gene miRNA microarray (Human_miRNA_17v1.0.0, Toray Industries) can mount >1500 miRNAs based on the Human miRNA Version 17 of MiRbase (http://microrna.sanger.ac.uk/). The microarray was scanned, and the images obtained were numerated using a 3D-GeneH scanner 3000 (Toray Industries). The expression level of each miRNA was globally normalized using the background-subtracted signal intensity of the entire set of miRNAs in each microarray. The obtained microarray images were analyzed using GenePix Pro^TM^ (Molecular Devices, Sunnyvale, CA). To identify candidate miRNAs, we focused on the miRNAs with an ID number less than 2000. This is because little is known concerning biological functions of miRNAs with an ID number more than 2000.

### Quantification of miRNA by qRT-PCR

The amounts of miRNAs were quantified by qRT-PCR using a Human TaqMan MicroRNA Assay Kit (Applied Biosystems, Foster City, CA). The reverse transcription reaction was conducted with a TaqMan MicroRNA Reverse Transcription Kit (Applied Biosystems) in 5 μl of solution containing 1.67 μl of extracted RNA, 0.05 μl of 100 mM dNTPs, 0.33 μl of Multiscribe Reverse Transcriptase (50 Uµl^−1^), 0.5 μl of 10× Reverse Transcription Buffer, 0.06 μl of RNase inhibitor (20 Uµl^−1^), 1 μl of gene-specific primer (hsa-miR-451, Assay ID: 001105; hsa-miR-126, Assay ID: 000450; hsa-miR-145, Assay ID: 000467; hsa-miR-146b-5p, Assay ID: 001097; hsa-miR-491-5p, Assay ID: 001630; hsa-miR-107, Assay ID: 000443; cel-miR-39, Assay ID: 000200; and RNU6B, Assay ID: 001093), and 1.39 μl of nuclease-free water. To synthesize cDNA, reaction mixtures were incubated at 16 °C for 30 min, at 42 °C for 30 min, and at 85 °C for 5 min, and were then held at 4 °C. Next, 0.67 μl of cDNA was amplified using 5 µl of TaqMan 2× Universal PCR Master Mix with no AmpErase UNG (Applied Biosystems), 0.5 µl of gene-specific primers/probes, and 3.83 µl of nuclease-free water in a final volume of 10 µl. qPCR was run on a StepOnePlus PCR system (Applied Biosystems), and reaction mixtures were incubated at 95 °C for 10 min, followed by 40 cycles of 95 °C for 15 sec and 60 °C for 1 min. Cycle threshold (Ct) values were calculated with StepOne Software v2.0 (Applied Biosystems).

As previously reported^16^, we used an approach for data normalization based on spiking the samples with a synthetic RNA oligonucleotide, cel-miR-39, which does not exist in the human genome. *C. elegans* cel-miR-39 was purchased as a custom-made RNA oligonucleotide (*Qiagen*, Valencia, CA). The oligo used for spiking, as a mixture of 25 fmol of oligonucleotide in a total water volume of 5 µl, was introduced after the addition of 2X Denaturing Solution (Ambion) to the plasma or serum sample to avoid degradation by endogenous plasma RNases. As a control for each RNA sample, cel-miR-39 was used for the TaqMan qRT-PCR assays (Applied Biosystems) as described above. We normalized the data across samples using the 2^−ΔΔCt^ method relative to cel-miR-39. However, the expression of miRNAs from tissue samples and cultured cells was normalized using the 2^−ΔΔCt^ method relative to U6 small nuclear RNA (RNU6B). ΔCt was calculated by subtracting the Ct values of cel-miR-39 or RNU6B from those of the miRNAs of interest. ΔΔCt was then calculated by subtracting the mean of ΔCt of healthy volunteer plasma or normal pancreatic tissue from the ΔCt of PCa plasma or tissues. The change in gene expression was calculated using the 2^−ΔΔCt^ method^101, 102^.

### Culture of PCa cell lines

The PCa cell lines PK-45H, PANC-1, MIA PaCa-2, and KP4-1 were purchased from RIKEN Cell Bank (Tsukuba, Japan) and cultured in Roswell Park Memorial Institute 1640 medium (Sigma, St. Louis, MO) or Dulbecco’s Modified Eagle Medium (Nacalai, Japan) supplemented with 10% fetal bovine serum (Trace Scientific, Melbourne, Australia). All cells were cultured in 5% carbon dioxide at 37 °C in a humidified chamber.

### Transfection of PCa cells with miRNA mimics

For the overexpression of miR-107, the miR-107 mimic (Assay ID: MC10056) or negative control mimic miRNA (mirVana miRNA mimic Negative Control #1), both of which were selected from the mirVana miRNA mimic panel (Ambion), was used to transfect the PK-45H, PANC-1, MIA PaCa-2, and KP4-1 cells at a final concentration of 12 μM by using Lipofectamine RNAiMAX (Invitrogen) according to the manufacturer’s instructions. After 72 h, the overexpression of miR-107 was confirmed by qRT-PCR using a Human TaqMan MicroRNA Assay Kit (Applied Biosystems).

### Proliferation assay and cell cycle analysis

To measure cell growth, the number of viable cells at various time points after transfection was assessed by the colorimetric water-soluble tetrazolium salt assay (Cell Counting Kit 8; Dojindo Laboratories, Kumamoto, Japan). Cell viability was determined by reading the optical density at 450 nm. The cell cycle was evaluated 72 h after transfection by fluorescence-activated cell sorting (FACS), as described elsewhere^103^. For the FACS analysis, harvested cells were fixed in 70% cold ethanol and treated with RNase A and propidium iodide. Samples were analyzed on a Becton Dickinson Accuri™ C6 Flow Cytometer (Becton Dickinson, San Jose, CA, USA).

### Colony-formation assays

The miR-107 or negative control mimic was introduced into PCa cells. The expression of the miR-107 mimic in transfected cells was confirmed by qRT-PCR. After 2 weeks of incubation, the cells were fixed with 100% methanol and stained with crystal violet.

### Apoptotic cell analysis

As a control, non-transfected cells were treated with staurosporine for 24 h. At 72 h after transfection, the miRNA mimic-transfected cells were harvested and stained with fluorescein isothiocyanate-conjugated annexin V and phosphatidylinositol using an Annexin V Kit (Beckman Coulter, Brea, CA). A Becton Dickinson Accuri™ C6 Flow Cytometer was used to analyze the proportion of apoptotic cells.

### Western blot analysis

Anti-ACTB, anti-p21, and anti-Notch2 antibodies were purchased from Santa Cruz Biotechnology (Santa Cruz, CA) and Cell Signaling Technology (Cell Signaling Technology, USA). Cells were lysed, and their proteins were extracted using M-PER® Mammalian Protein Extraction Reagent (Thermo Scientific, USA).

### Isolation of exosomes from plasma

Exosomes were extracted from plasma using a miRCURY Exosomes Isolation Kit-serum and plasma (EXIQON). Thrombin was added to the plasma, and the supernatants were collected after centrifugation at 10,000 g for 5 min. Precipitation buffer was added to the supernatants, and exosome pellets were collected by centrifugation at 500 g for 5 min after incubation for 60 min at 4 °C.

### miRNA isolation from exosomes

miRNAs were isolated from exosomes using a mirVana PARIS Kit (Ambion, Austin, TX) and were eluted into 100 Ul of heated elution solution according to the manufacturer’s protocol.

### Animal experimental protocol

For the *in vivo* model, PCa cells (5 × 10^6^ MIA PaCa-2) were subcutaneously inoculated on one side of the ventral surface in the lower flank region of SCID mice. Treatment began at 7 days after tumor cell implantation. Either 1 nmol of the control miRNA mimic or 1 nmol of the miR-107 mimic with AteloGene Local Use Quick gelation (Koken, Co., Tokyo, Japan) was subcutaneously injected around the tumor once a week for 4 weeks, according to the manufacturer’s protocol. The tumor volume was calculated according to the formula V = A × B^2^/2 (mm^3^), where A is the largest diameter (mm), and B is the smallest diameter (mm). At 29 days after tumor cell implantation, the mice were sacrificed and blood samples were collected for further analysis. In another *in vivo* model, PCa cells (5 × 10^6^ MIA PaCa-2) were subcutaneously inoculated on one side of the ventral surface in the lower flank region of SCID mice. Three weeks after tumor cell implantation, the mice were sacrificed and tissue samples from such organs as the liver and spleen were collected to compare the expression of miR-107 between tumor-bearing mice and mice without tumors. The animal protocol was approved by the Institutional Animal Care and Use Committee of Kyoto Prefectural University of Medicine and all experiments were conducted strictly in accordance to the National Institute of Health Guide for Care and Use of Laboratory Animals.

### Statistical analysis

For the miRNA array-based analyses, the signal intensity ratio of each plasma miRNA was calculated as the signal intensity ratio of PCa patients to healthy volunteers. The Mann–Whitney U test and the t-test for unpaired data were performed for comparing plasma or tissue sample data. The Wilcoxon test was used to compare the paired plasma samples obtained before and 1 month after pancreatectomy and the paired tumor and normal tissue samples. The Chi-square test or Fisher’s exact probability test was used to evaluate correlations between the plasma miRNA levels and clinicopathological factors. A *P*-value < 0.05 was considered statistically significant. Receiver-operating characteristic (ROC) curves and area under the ROC curve (AUC) values were used to assess the feasibility of using plasma miRNA levels as a diagnostic tool for detecting PCa. The ROC curve was created by plotting the sensitivity against the false positive rate (1- specificity) at various threshold settings. The Youden index was used to determine the cut-off value for the plasma miRNA levels^45^. For the survival rate analysis, Kaplan–Meier survival curves were constructed for groups based on univariate predictors, and differences between the groups were analyzed with the log-rank test or the Wilcoxon test. Univariate and multivariate survival analyses were performed using the likelihood ratio test of the stratified Cox proportional hazards model. A *P*-value < 0.05 was considered statistically significant.

### Data Availability

All data generated or analysed during this study are included in this published articles. The microarray data from this publication have been submitted to the GEO database (http://www.ncbi.nlm.nih.gov/geo/) and assigned the identifier “GSE92424”.

## Electronic supplementary material

Supplementary Materials
