# Supplementary material for: Depleted tumor suppressor miR-107 in plasma relates to tumor progression and is a novel therapeutic target in pancreatic cancer
Source: Sci Rep. 2017 Jul 18;7:5708. doi: 10.1038/s41598-017-06137-8 (PMC5515843; doi:10.1038/s41598-017-06137-8)
Supplement: Supplementary file 1 — Supplementary Materials [file 41598_2017_6137_MOESM1_ESM.doc]

**Supplementary Materials**

**Depleted tumor suppressor miR-107 in plasma relates to tumor progression and is a novel therapeutic target in pancreatic cancer**

Taisuke Imamura^1,+^, Shuhei Komatsu^1,+,*^, Daisuke Ichikawa^1^, Mahito Miyamae^1^, Wataru Okajima^1^, Takuma Ohashi^1^, Jun Kiuchi^1^, Keiji Nishibeppu^1^, Hirotaka Konishi^1^, Atsushi Shiozaki^1^, Ryo Moriumura^1^, Hisashi Ikoma^1^, Toshiya Ochiai^1^, Kazuma Okamoto^1^, Hiroki Taniguchi^2^, and Eigo Otsuji^1^

^1^Division of Digestive Surgery, Department of Surgery, Kyoto Prefectural University of Medicine, 465 Kajii-cho, Kawaramachihirokoji, Kamigyo-ku, Kyoto, 602-8566, Japan

^2^Department of Surgery, Kyoto Second Red Cross Hospital, 355-5 Kamanzadoori Marutamachi Haruobicho, Kamigyo-ku, 602-8026, Kyoto, Japan

^+^ These authors (T.I. and S.K.) equally contributed to this work.

****Corresponding author:*** Shuhei Komatsu, MD, PhD. Phone: +81-75-251-5527, Fax: +81-75-251-5522, E-mail: [skomatsu@koto.kpu-m.ac.jp](mailto:skomatsu@koto.kpu-m.ac.jp)


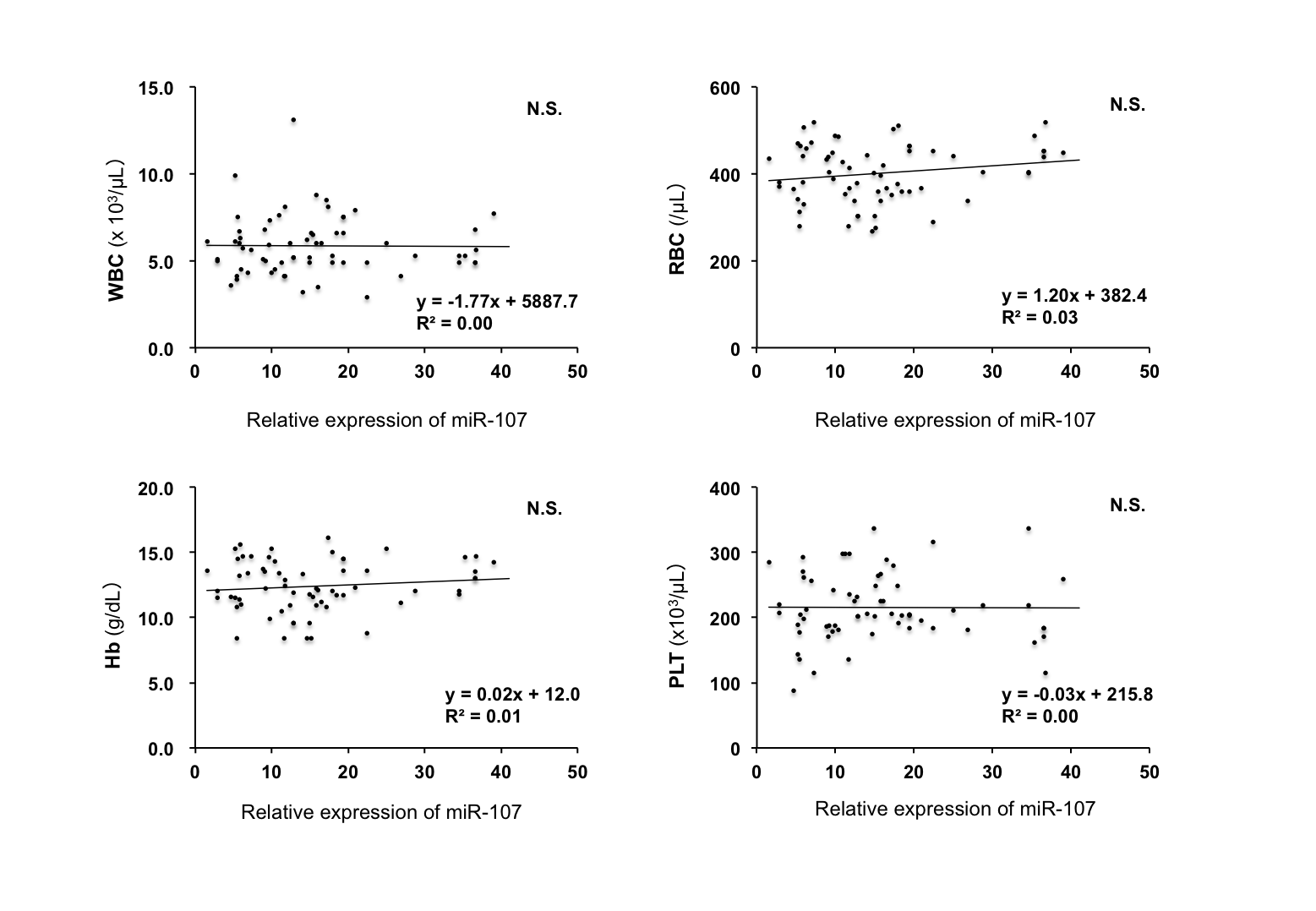


**Supplementary Figure S1 Evaluation of the correlation between the plasma level of miR-107 and peripheral blood cells.**

The numbers of leucocytes, erythrocytes, and platelets and the hemoglobin concentrations in the peripheral blood of PCa patients were analyzed based on the plasma levels of miR-107 using Spearman’s correlation test. Each dot indicates an individual patient. NS: not significant. WBC: white blood cell. RBC: red blood cell. Hb: hemoglobin. PLT: platelet.


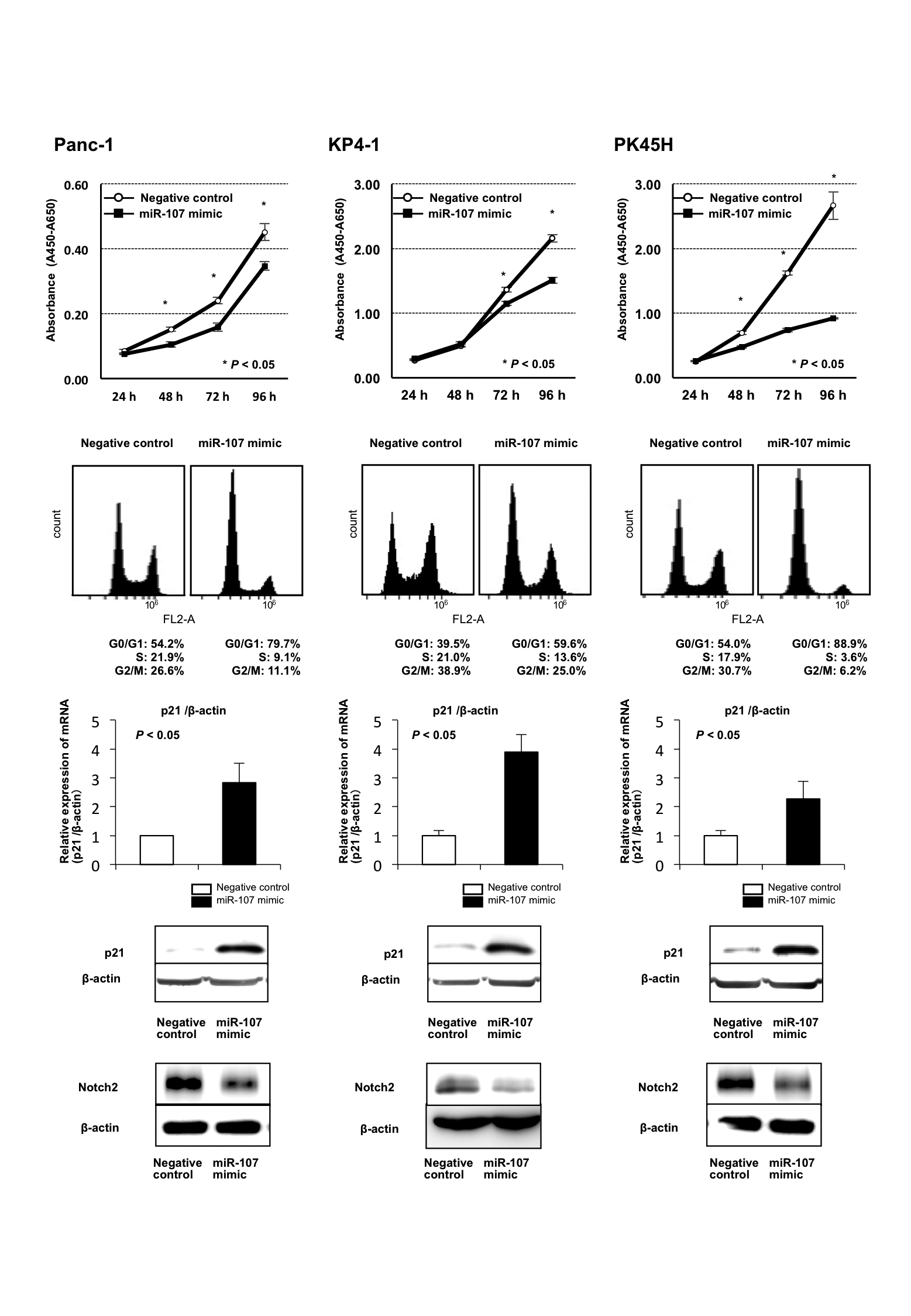


**Supplementary Figure S2 Confirmation of the tumor suppressor function of miR-107 in multiple other PCa cell lines.**

Proliferation was significantly suppressed in cells transfected with the miR-107 mimic compared with cells transfected with the negative control mimics. The FACS analysis demonstrated that miR-107 mimic transfection resulted in an accumulation of cells in the G0–G1 phase compared with control miRNA mimic transfection. In all these pancreatic cancer cell lines, the p21 mRNA and protein levels were increased and the Notch2 protein level was reduced at 72 h after miR-107 mimic transfection.

| **Supplementary Table S1. Patient characteristics** | | | |
| --- | --- | --- | --- |
|  | Variables | n |  |
|  | Total | 100 |  |
|  | Gender |  |  |
|  | Male | 52 |  |
|  | Female | 48 |  |
|  | Age |  |  |
|  | < 65 | 32 |  |
|  | ≥ 65 | 68 |  |
|  | Tumor location |  |  |
|  | Ph ^a^ | 39 |  |
|  | Pbt ^b^ | 35 |  |
|  | Ph or Pbt with distant metastasis | 26 |  |
|  | T stage |  |  |
|  | T1 / T2 | 8 |  |
|  | T3 / T4 / TX | 92 |  |
|  | N stage |  |  |
|  | N0 | 34 |  |
|  | N1 / NX | 66 |  |
|  | M stage (TNM) |  |  |
|  | M0 | 70 |  |
|  | M1 / MX | 30 |  |
|  | Treatment |  |  |
|  | Resectable | 74 |  |
|  | Unresectable | 26 |  |
| ^a^Pancreatic head, ^b^pancreatic body or tail. | | | |
